# Special Issue: Host Cell–Virus Interaction

**DOI:** 10.3390/v14030615

**Published:** 2022-03-16

**Authors:** Anupam Mukherjee, Parikshit Bagchi

**Affiliations:** 1 Division of Virology, ICMR-National AIDS Research Institute, Pune 411026, Maharashtra, India; 2 Department of Cell and Developmental Biology, University of Michigan Medical School, Ann Arbor, MI 48109, USA

As rightly put by Nobel Laureate Joshua Lederberg, “the single biggest threat to man’s continued dominance on the planet is the Virus”. What this respected microbiologist declared long ago still holds true in the present day. Every virus is capable of hijacking a host cell—be it a plant, animal, insect or even a bacterial cell—to replicate and survive at the cost of its host. It is the interactions of the virus with the host cell that hold the deepest and darkest secrets of the virus, the unraveling of which opens up multiple avenues in antiviral research. Studies of host cell–virus interaction not only provide crucial information to virologists, but also attract and impact the research of cell biologists and immunologists. Host–virus interaction studies may lead to the discovery of novel host cell protein functions that contribute toward understanding connections with other related viruses. The regulation of cellular immune responses is another significant component of viral pathogenesis. In this Special Issue, we focused on research covering the strategies that viruses exploit for successful infection of host cells, and highlighted the counteractive measures employed by host cells in order to win the war against the viruses.

In this Special Issue, we received submissions from every corner of the world, spanning diverse fields related to host cell–virus interactions. A total of ten original research articles and three review articles were accepted and published. Amid the COVID-19 pandemic situation, we will first highlight the article by Caillet-Saguy and Wolff, which focused on the identification of cellular PDZ-containing proteins using angiotensin-converting enzyme 2 (ACE2) during SARS-CoV-2 infection [1]. This research communication reported that PDZ/PBM interactions with ACE2 may be beneficial for virus entry, and affirmed that neuronal proteins, e.g., of the SHANK and MAST families, may be involved in the neurological symptoms of COVID-19.

TGF-β and its dual characteristics are consistently implicated in either impeding HIV infection by counteracting HIV-LTR or increasing viral burden and promotion of HIV-1 latency. Thus, Gokavi et al. demonstrated the role of TGF-β as an effective negative regulator of HIV expression in vitro [2]. The study also revealed the role of microRNA-155 in the release of infectious HIV particles while neutralizing TGF-β.

Human T-cell leukemia virus (HTLV) is a human retrovirus that possesses proteins capable of oncogenesis. In two different studies, it was identified that Rex—the HTLV-1 (type 1) RNA-binding protein—interferes in the diverse pathways of host T cells to regulate viral replication (Nakano et al. [3]), and attacks nonsense-mediated mRNA decay or NMD cascade during HTLV-1 mRNA trafficking to protect the viral genomic mRNA (Nakano et al. [4]). In contrast, HTLV-2 (type 2) anti-sense protein or APH-2 has been examined due to its interference in HIV-1 replication and its negative influence on different stages of the HIV-1 lifecycle (Londhe and Kulkarni) [5].

Macrophages are the sentinels of the immune system that have certain effector functions such as antigen presentation, phagocytosis and release of pro/anti-inflammatory cytokines while exposed to viral pathogens. In certain viral infections, interleukin (IL)-1β- and IL-18-mediated activation of the inflammasome is commonly observed. Hence, Eisfeld et al. described the mechanistic role of SARS-CoV-1, SARS-CoV-2, HCMV and HCV viral glycoproteins as innate immunity triggers, in vitro, through activation of the NLRP3 inflammasome and the induction of lytic cell death via Gasdermin D or GSDMD-dependent pyroptosis [6].

Direct-acting antiviral (DAA) drugs are highly effective in curing hepatitis C virus (HCV) infection but they do have known risks, including reactivation of hepatitis B virus and several critical drug–drug interactions. Moreover, numerous challenges still remain in HCV treatment, such as barriers to screening, linkage to care, access to medication, and high pharmaceutical costs. In a study conducted on Egyptian HCV-infected patients receiving DAAs, a direct association was shown between prognostic markers such as interlukin-13, vitamin D, and the expression of miR-135a with treatment failure and hepatocellular carcinoma (HCC) development (Ali et al.) [7].

Rotaviruses are the most prominent etiologic agents of infantile gastroenteritis. The activation of immune responses is the key factor for host defense against any viral infections and rotavirus is no exception. This important aspect is elucidated by Sarkar et al. through demonstration of the role of Viperin in limiting rotavirus egress by controlling NSP4-mediated apoptosis, as well as highlighting the novel antirotaviral action of this multifunctional, interferon-inducible protein [8].

Flaviviruses are involved in severe neurological complications and, hence, are one of the most important agents affecting human health globally. Most flaviviruses are primarily transmitted by a bite from an infected arthropod, mosquito or ticks. In this Special Issue, the articles by Breitkopf et al. and Reis et al. revealed the impact of tick-borne flavivirus-mediated immune signaling response in astrocytes against viral infection and neuroinflammation, and the αVβ3 integrin-mediated enhancement of flavivirus replication, respectively [9,10]. Likewise, the review article by Nanaware et al. on Dengue virus infection, another flavivirus, discusses the different stages of host protein exploitation by the virus in the course of infection as well as the host’s counteracting mechanism [11].

Herpes stromal keratitis (HSK) is the key infection that is driven by the presence of HSV-1 antigen within the stroma, which can lead to progressive corneal scarring and permanent visual impairment. Though existing treatments are effective for those suffering with HSK, there is an urgent need for novel treatment regimes to address the increasing disease burden. The review article by Greenan et al. confers similar importance to current understanding of HSV-1 infection and discusses prospective novel treatment options including microRNA-, TLR-, mAb-, and aptamer-based therapeutics [12].

Finally, the review by Elkhaligy et al. provides an overview of known viral mimicry through short linear motifs (SLiMs), and highlights the importance of viral SLiMs at selected steps of multiple viruses’ life cycles [13]. SLiMs are small segments of proteins, 3 to 10 amino acids long, with a specific cellular function, and act as the functional signatures for understanding protein–protein interactions in any organism. The authors concluded that specific SLiMs may affect the virulence and pathogenicity of certain viruses such as flaviviruses, influenza viruses, Chikungunya virus, adenovirus, foot and mouth disease virus, Epstein–Barr virus, SARS-CoV-2, HCV, HPV18, HIV-1, etc.

The contributors of this Special Issue have highlighted the latest advancements in the field of host cell–virus interactions. Together, this information will undoubtedly improve our understanding of the virus–host interface and host-targeting antiviral approaches. As the Guest Editors of this Special Issue, we express our sincere gratitude to all the authors who contributed their valuable findings to “Host Cell–Virus Interaction”.
